# Obesity-Associated Cancers: A United States Cancer Statistics (USCS) Database Analysis

**DOI:** 10.7759/cureus.84610

**Published:** 2025-05-22

**Authors:** Jane N Nwafor, Alexander S Figueroa, Okelue E Okobi, Gift Ojukwu, Edamisan J Fanegan, Robert Nyamekye-Affel, Blessing O Oyewole, Oluwatobiloba Omotunde, Grace N Mamah, Nneka Muoghalu

**Affiliations:** 1 Internal Medicine, University of the District of Columbia, Washington, DC, USA; 2 Medicine, University of the East Ramon Magsaysay Memorial Medical Center (UERMMMC) College of Medicine, Quezon City, PHL; 3 Family Medicine, IMG Research Academy and Consulting LLC, Homestead, USA; 4 Family Medicine, Larkin Community Hospital Palm Springs Campus, Hialeah, USA; 5 Family Medicine, Lakeside Medical Center, Belle Glade, USA; 6 General Practice, Leeds Teaching Hospitals NHS Trust, Leeds, GBR; 7 Post Heart Catheterisation, RWJBarnabas Health, Newark, USA; 8 Internal Medicine, St. Patrick's Hospital, Kumasi, GHA; 9 Family Medicine, Northfield Family Health, Canada, CAN; 10 Internal Medicine, American International School of Medicine, Atlanta, USA; 11 Family and Community Medicine, University of Nigeria College of Medicine, Enugu, NGA; 12 Public Health, Liverpool School of Tropical Medicine, University of Liverpool, Liverpool, GBR; 13 Internal Medicine, University College Hospital, Ibadan, NGA

**Keywords:** database, obesity, obesity-associated cancer, retrospective study, uscs

## Abstract

Background: Obesity is a well-established risk factor for various cancers, contributing to significant public health burdens. Disparities in obesity-associated cancer incidence exist across racial, age, and geographic groups, necessitating targeted prevention and intervention strategies.

Objective: The aim of this study is to analyze the incidence rates of obesity-associated cancers across different racial, age, and geographic groups in the United States from 2017 to 2021, identifying key disparities to inform public health interventions.

Methods: A retrospective analysis of cancer incidence data from national registries was conducted. Age-adjusted incidence rates (per 100,000 population) were calculated across racial/ethnic groups, age cohorts, and US states. Descriptive statistics and confidence intervals were used to assess disparities.

Results: Black, non-Hispanic individuals had the highest obesity-associated cancer incidence (184.8 per 100,000), followed by American Indian/Alaska Native populations (179.3 per 100,000). Incidence rates increased with age, peaking at 75-79 years (788.7 per 100,000 overall). Geographically, Midwestern and Southern states exhibited higher incidence rates, with West Virginia reporting the highest (188.3 per 100,000) and Nevada the lowest (149.5 per 100,000). These findings highlight significant racial, age, and regional disparities.

Conclusion: The study underscores the need for targeted public health strategies, including enhanced screening, culturally tailored interventions, and policy-driven approaches to address obesity and its related cancer risks. Future research should explore individual-level risk factors and effective interventions to promote equitable healthcare access and improved cancer outcomes.

## Introduction

Obesity, defined as a body mass index (BMI) of 30 kg/m² or higher, is a major public health concern worldwide, contributing to a significant burden of chronic diseases, including cardiovascular conditions, metabolic disorders, and various malignancies [1]. Cancer is characterized by the uncontrolled proliferation of abnormal cells capable of invading tissues and metastasizing to distant organs, representing a heterogeneous group of diseases with diverse etiologies [2,3]. Over the past five decades (from 1970 to present), the prevalence of obesity has risen substantially, paralleling an increase in obesity-associated cancers, with excess body weight being implicated in the pathogenesis of multiple cancer types, including colorectal, postmenopausal breast, endometrial, pancreatic, renal, and oesophageal adenocarcinomas [2,3].

The epidemiology of obesity-associated cancer has been extensively studied, demonstrating a growing burden in both developed and developing nations. About 4-8% of all cancers are attributed to obesity [4]. Obesity is a risk factor for several primary cancers, including post-menopausal breast, colorectal, endometrial, kidney, oesophageal, pancreatic, liver, and gallbladder cancer. Excess body fat results in an approximately 17% increased risk of cancer-specific mortality [4]. Continuously rising trends in obesity-related malignancies render this disease spectrum a public health priority. Worldwide, the burden of cancer attributable to obesity, expressed as a population-attributable fraction, is 11.9% in men and 13.1% in women [5]. The US Centers for Disease Control and Prevention (CDC) released a new report on cancer and obesity last week, highlighting that cancers associated with overweight and obesity, including thyroid, liver, kidney, and ovarian cancer, constitute 40% of cancers diagnosed in the USA, with over 630,000 diagnoses in 2014 alone [6].

Obesity contributes to carcinogenesis through multiple biological mechanisms, including chronic inflammation, insulin resistance, and alterations in sex hormone metabolism. Adipose tissue acts as an endocrine organ, secreting pro-inflammatory cytokines such as interleukin-6 (IL-6) and tumor necrosis factor-alpha (TNF-α), which promote cellular proliferation and inhibit apoptosis [7]. Hyperinsulinemia, a hallmark of obesity-related metabolic dysfunction, enhances insulin-like growth factor 1 (IGF-1) signaling, further driving tumor growth [8]. Additionally, increased estrogen production from adipose tissue in obese individuals is linked to heightened risks of hormone-dependent cancers, such as breast and endometrial malignancies [9]. The interplay between obesity, the tumor microenvironment, and genetic susceptibility underscores the complexity of obesity-associated cancer development and progression [10].

The United States Cancer Statistics (USCS) database is a comprehensive surveillance system that aggregates cancer incidence and mortality data from central cancer registries across all states and territories [11]. Jointly managed by the CDC and the National Cancer Institute (NCI), the USCS database provides high-quality, standardized data for epidemiological research [11]. This study utilizes data from 2017 to 2021, capturing recent trends in obesity-related cancer incidence. Variables of interest include age, sex, race/ethnicity, cancer type, and geographical distribution. The robustness of USCS data ensures a reliable assessment of obesity-associated cancer patterns, facilitating evidence-based policy recommendations.

The primary objective of this study is to analyze the incidence and trends of obesity-associated cancers in the USA from 2017 to 2021 using the USCS data. Specifically, we aim to evaluate temporal trends in obesity-related cancer incidence, assess demographic and geographic variations in cancer burden, and identify potential disparities in obesity-associated cancer outcomes. By elucidating these trends, our findings will contribute to the development of targeted public health interventions aimed at reducing obesity-related cancer risks. Additionally, this research seeks to inform healthcare policies, emphasizing the need for early detection and preventive strategies tailored to high-risk populations.

## Materials and methods

Data source and study design

This study utilizes data from the United States Cancer Statistics (USCS) database, which compiles cancer incidence and mortality data from population-based cancer registries across the USA. The study follows a retrospective cohort design, examining trends in obesity-associated cancers between 2017 and 2021. Data from multiple registries were harmonized to ensure consistency in case definitions and reporting.

Study participants and questionnaires

The study population includes all individuals diagnosed with obesity-associated cancers between 2017 and 2021, as defined by the CDC and the International Agency for Research on Cancer (IARC). Thirteen cancer types with established epidemiological links to obesity were included, and cases were identified using the International Classification of Diseases for Oncology, Third Edition (ICD-O-3) topography and histology codes, following CDC/USCS conventions. Only invasive cases were included. For breast cancer, we restricted the sample to women aged 50 years or older to approximate postmenopausal status. Full definitions and codes used for each cancer type are described under the Variables of Interest section. No direct patient interviews or questionnaires were conducted.

Data collection and quality assurance

Cancer registries within the USCS database collect information through standardized protocols, incorporating data from hospital records, pathology reports, and state health departments. Quality assurance measures, including duplicate record checks, coding verification, and validation studies, were implemented to maintain data integrity and reliability. Data completeness was ensured using the USCS quality standards. Cases with missing values for key variables, such as age, sex, primary site, and diagnosis year, were excluded. Less than 3% of cases were missing age or sex, and less than 5% were missing race/ethnicity, which falls within acceptable USCS thresholds. No imputation was conducted; a complete-case analysis approach was used throughout.

Variables of interest

Key variables include demographic characteristics (age, sex, race/ethnicity), cancer type, tumor staging, and geographic distribution. Obesity-associated cancers were defined based on IARC and CDC classifications, covering 13 cancer types: adenocarcinoma of the esophagus; postmenopausal breast cancer; cancers of the colon and rectum; endometrial, gallbladder, gastric cardia, kidney (renal cell), liver, ovarian, pancreatic, and thyroid cancers; and meningioma and multiple myeloma. These were identified using ICD-O-3 topography and histology codes consistent with USCS and surveillance, epidemiology, and end results (SEER) protocols. A complete list of codes and inclusion criteria was applied to ensure reproducibility.

Obesity-associated cancers were defined in accordance with the IARC and CDC/USCS conventions. We included the 13 cancer types for which there is sufficient evidence of association with excess adiposity: adenocarcinoma of the esophagus; postmenopausal breast cancer; cancers of the colon and rectum; endometrial cancer; gallbladder cancer; gastric cardia cancer; kidney (renal cell) cancer; liver cancer; ovarian cancer; pancreatic cancer; thyroid cancer; meningioma; and multiple myeloma. Only invasive cases were included. To approximate postmenopausal status, breast cancer cases were limited to women aged ≥50 years. Each cancer type was identified using the ICD-O-3 site and histology codes. Esophageal adenocarcinoma was defined using site codes C15.0-C15.9 and adenocarcinoma histology codes 8140/3-8576/3. Colorectal cancers included site codes C18.0-C18.9 (colon) and C19.9-C20.9 (rectum) with adenocarcinoma histology codes. Endometrial cancers were defined as C54.1 (corpus uteri) with histology codes including 8380/3. Other sites included C23.9 (gallbladder), C16.0 (gastric cardia), C64.9 (kidney), C22.0-C22.9 (liver), C56.9 (ovary), C25.0-C25.9 (pancreas), and C73.9 (thyroid), each linked to appropriate malignant histologies. Meningiomas were identified using central nervous system topography codes C70.x-C72.x and World Health Organization morphology codes for meningioma. Multiple myeloma was defined using hematopoietic site codes C42.0-C42.1 with histology code 9731/3. These operational definitions follow standard cancer registry guidelines to support the reproducibility of our case selection.

Data analysis and statistical methods

Descriptive statistics were used to summarize baseline characteristics of the study population. Incidence rates were age-adjusted using the direct method and the 2000 US standard population. Population denominators were derived from the US Census Bureau’s bridged-race estimates, as adapted by the CDC and NCI for USCS reporting. These estimates accounted for sex-, age-, and race/ethnicity-specific differences at the national and state levels. To compare incidence trends across subgroups, one-way analysis of variance (ANOVA) was used. This method tested whether mean rates differed significantly by race/ethnicity, sex, and cancer type. The assumptions of normality and equal variance were evaluated, and log transformations were applied where appropriate. All analyses were performed using Statistical Product and Service Solutions (SPSS, version 30; IBM SPSS Statistics for Windows, Armonk, NY), with statistical significance set at 0.05.

Ethical considerations

This study utilizes publicly available, de-identified data from the USCS database, ensuring compliance with ethical research standards. No direct patient contact or identifiable information was involved, exempting the study from institutional review board (IRB) approval. Data handling procedures adhered to federal and institutional guidelines for confidentiality and research integrity.

## Results

The results highlight significant disparities in obesity-associated cancer incidence across racial, age, and geographic groups, emphasizing the influence of genetic, socioeconomic, and environmental factors on cancer risk and prevalence.

Based on racial disparities in obesity-associated cancer incidence

**Figure 1:**
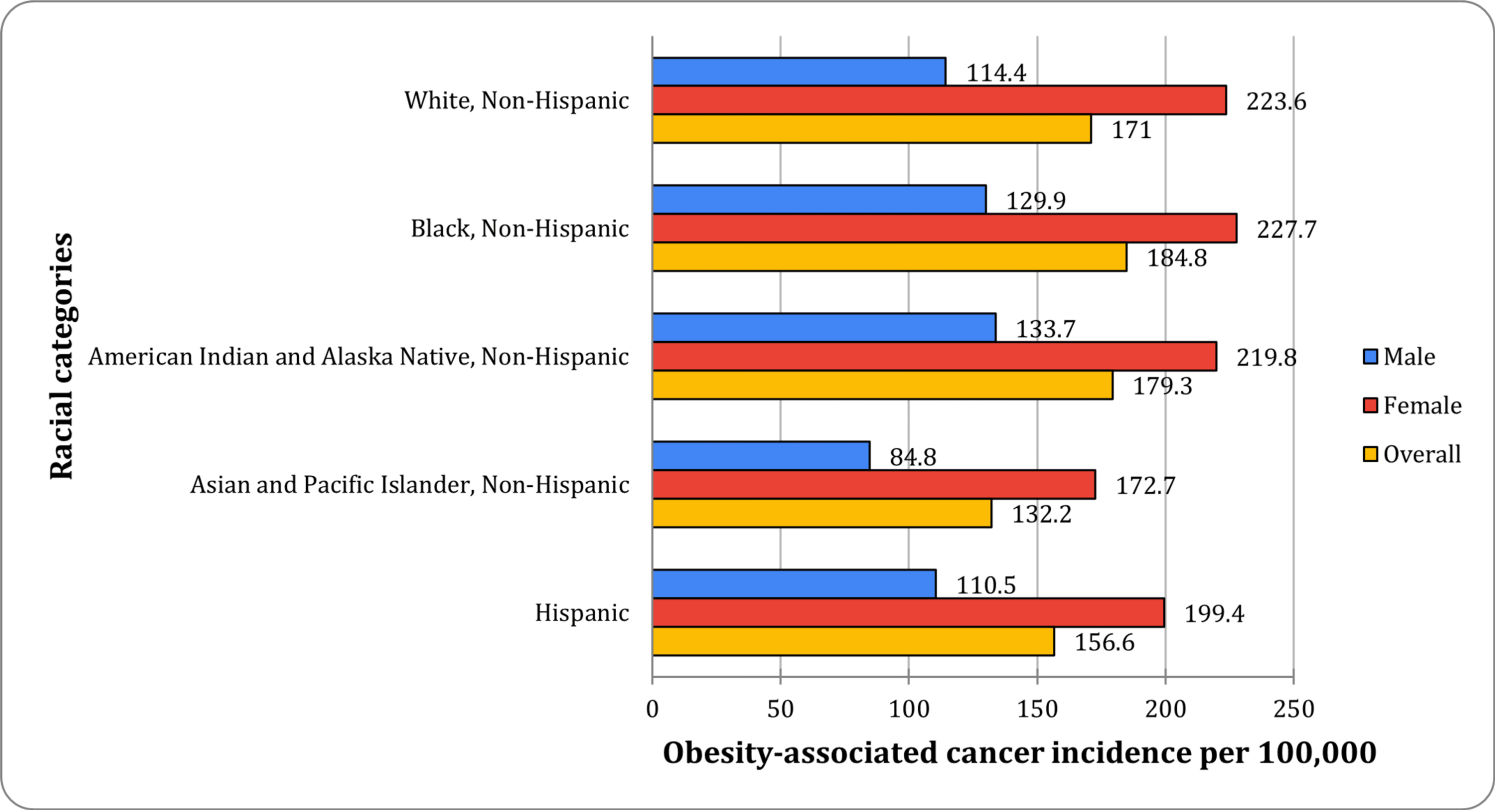
Racial disparities in obesity-associated cancer incidence Data source: United States Cancer Statistics (2018–2022) [4]

The analysis of age-adjusted incidence rates for obesity-associated cancers, based on 2018-2022 data from the USCS database, revealed substantial racial disparities (p < 0.05). Black, non-Hispanic individuals exhibited the highest age-adjusted incidence rate at 184.8 cases per 100,000 population (95% CI: 184.2-185.4), marking them as the group with the most significant burden of obesity-associated cancers. American Indian and Alaska Native, non-Hispanic individuals followed with a rate of 179.3 cases per 100,000 (95% CI: 176.9-181.6). White, non-Hispanic individuals reported 171.0 cases per 100,000 (95% CI: 170.7-171.2), while Hispanic individuals exhibited a lower incidence of 156.6 cases per 100,000 (95% CI: 156.1-157.2). The lowest incidence was observed among Asian and Pacific Islander, non-Hispanic individuals at 132.2 cases per 100,000 (95% CI: 131.5-132.9). Figure 1 illustrates these racial disparities using incidence data sourced from the USCS database (2018-2022) [4].

These findings underscore persistent racial disparities in cancer incidence, necessitating further investigation into underlying genetic, lifestyle, and socioeconomic factors that may contribute to these variations. Table 1 provides a detailed breakdown of age-adjusted incidence rates (per 100,000) with 95% confidence intervals (CI) for obesity-associated cancers across racial groups, stratified by sex and age group, based on USCS data [4].

**Table 1:** Age-adjusted incidence rates per 100,000 population of obesity-associated cancers by racial and ethnic groups, age, and sex Data source: United States Cancer Statistics, National Cancer Registries (2018–2022) [4] One-way ANOVA was conducted to assess the significance between variables.

Obesity-Associated Cancer Incidence per 100,000 Based on Racial and Ethnic Groups						
Characteristics	Total Number of Cases	Overall Incidence Rate per 100,000 Population (95% CI)	Age-Adjusted Incidence Rate per 100,000 Population (95% CI) for Males	Age-Adjusted Incidence Rate per 100,000 Population (95% CI) for Females	F value	P value
White, non-Hispanic	2,428,204	171.0 (170.7-171.2)	114.4 (114.1-114.6)	223.6 (223.3-224.0)	24.70	<0.05
Black, non-Hispanic	397,505	184.8 (184.2-185.4)	129.9 (129.2-130.7)	227.7 (226.9-228.6)		
American Indian and Alaska Native, non-Hispanic	24,632	179.3 (176.9-181.6)	133.7 (130.7-136.7)	219.8 (216.3-223.3)		
Asian and Pacific Islander, non-Hispanic	144,589	132.2 (131.5-132.9)	84.8 (83.9-85.6)	172.7 (171.6-173.8)		
Hispanic	352,887	156.6 (156.1-157.2)	110.5 (109.8-111.2)	199.4 (198.6-200.2)		
Age Group in Years						
<20	12,336	3.0 (2.9-3.1)	1.9 (1.8-2.0)	4.2 (4.1-4.2)	1.3216	0.2756
20-24	11,543	10.6 (10.4-10.8)	5.0 (4.8-5.2)	16.5 (16.2-16.9)		
25-29	20,762	18.5 (18.3-18.8)	9.3 (9.0-9.5)	28.2 (27.7-28.6)		
30-34	34,300	31.1 (30.8-31.5)	17.5 (17.2-17.9)	45.1 (44.6-45.7)		
35-39	52,009	48.4 (48.0-48.8)	31.6 (31.1-32.1)	65.6 (64.9-66.3)		
40-44	73,559	73.9 (73.4-74.5)	55.3 (54.7-56.0)	92.8 (91.9-93.6)		
45-49	113,717	112.9 (112.2-113.5)	94.5 (93.7-95.4)	131.4 (130.4-132.4)		
50-54	303,649	295.1 (294.0-296.1)	159.4 (158.3-160.5)	430.7 (428.9-432.5)		
55-59	399,929	371.7 (370.6-372.9)	221.7 (220.5-223.0)	517.8 (515.9-519.7)		
60-64	492,316	488.3 (487.0-489.7)	313.5 (311.9-315.0)	653.5 (651.3-655.7)		
65-69	537,335	627.8 (626.1-629.5)	416.9 (414.9-418.8)	819.0 (816.4-821.6)		
70-74	487,221	716.2 (714.1-718.2)	479.4 (477.0-481.8)	923.1 (920.0-926.3)		
75-79	359,734	788.7 (786.2-791.3)	561.8 (558.5-565.0)	974.0 (970.2-977.9)		
80-84	244,651	813.1 (809.8-816.3)	614.4 (610.1-618.7)	957.9 (953.3-962.5)		
85+	231,350	734.8 (731.8-737.8)	600.2 (595.7-604.9)	805.9 (802.1-809.8)		

A deeper analysis by sex revealed notable differences in incidence rates across racial groups. Among males, the highest incidence of obesity-associated cancers was recorded in American Indian and Alaska Native, non-Hispanic individuals, with 133.7 cases per 100,000 population (95% CI: 130.7-136.7). This was followed closely by Black, non-Hispanic males, who had an incidence of 129.9 (95% CI: 129.2-130.7), and White, non-Hispanic males, with a slightly lower incidence at 114.4 (95% CI: 114.1-114.6). Hispanic males exhibited a reduced incidence rate at 110.5 (95% CI: 109.8-111.2), while Asian and Pacific Islander, non-Hispanic males reported the lowest incidence at 84.8 (95% CI: 83.9-85.6).

In contrast, females demonstrated significantly higher incidence rates across all racial groups. The highest incidence was observed in Black, non-Hispanic females, with 227.7 cases per 100,000 population (95% CI: 226.9-228.6). White, non-Hispanic females closely followed with an incidence of 223.6 (95% CI: 223.3-224.0), while American Indian and Alaska Native, non-Hispanic females reported 219.8 cases per 100,000 (95% CI: 216.3-223.3). Hispanic females showed an incidence rate of 199.4 (95% CI: 198.6-200.2), and Asian and Pacific Islander, non-Hispanic females again had the lowest rate at 172.7 (95% CI: 171.6-173.8). These sex-based disparities emphasize the importance of sex-specific preventive strategies, particularly in racial groups with higher burdens of obesity-associated cancers.

Based on age group

**Figure 2:**
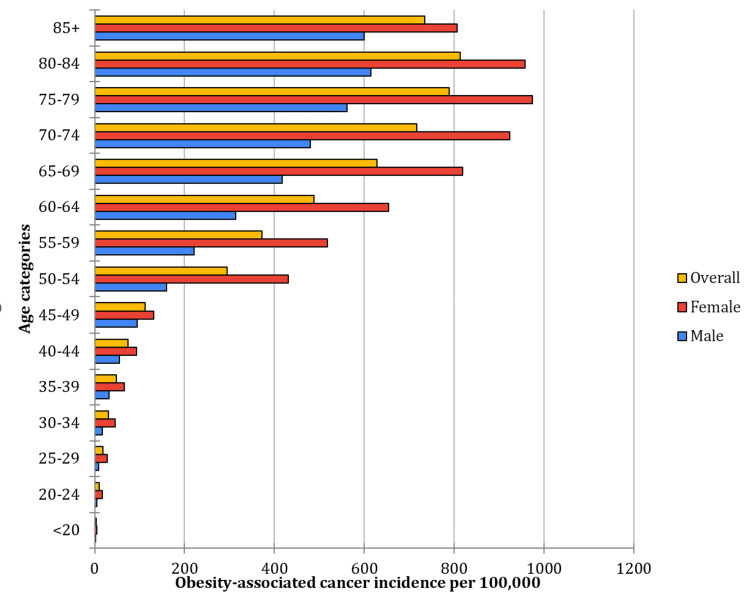
Age‑specific incidence rates per 100 000 population of obesity‑associated cancers by age group Data source: USCS National Cancer Registries (2018–2022) [4]

Analysis by age groups, using data from the USCS National Cancer Registries (2018-2022) [4], revealed a clear trend of increasing incidence rates per 100,000 population with advancing age (p > 0.05) (Figure 2). Individuals younger than 20 years demonstrated the lowest rates, with males at 1.9 cases per 100,000 (95% CI: 1.8-2.0), females at 4.2 (95% CI: 4.1-4.2), and an overall incidence rate of 3.0 (95% CI: 2.9-3.1). Incidence rates increased progressively across subsequent age groups, with individuals in the 50-54 age group experiencing significantly higher rates: 159.4 cases per 100,000 (95% CI: 158.3-160.5) in males, 430.7 (95% CI: 428.9-432.5) in females, and an overall incidence rate of 295.1 (95% CI: 294.0-296.1). Figure 2 shows the age-specific incidence of obesity-associated cancers [4].

The 60-64 age group exhibited further increases, with incidence rates of 313.5 cases per 100,000 in males (95% CI: 311.9-315.0), 653.5 (95% CI: 651.3-655.7) in females, and an overall rate of 488.3 (95% CI: 487.0-489.7). The peak incidence was observed in the 75-79 age group, reaching 561.8 cases per 100,000 (95% CI: 558.5-565.0) in males, 974.0 (95% CI: 970.2-977.9) in females, and an overall incidence of 788.7 (95% CI: 786.2-791.3). Further, a slight decline in incidence was noted in individuals aged 85 years and older, with males at 600.2 cases per 100,000 (95% CI: 595.7-604.9) and females at 805.9 (95% CI: 802.1-809.8), resulting in an overall rate of 734.8 (95% CI: 731.8-737.8). These findings highlight a strong correlation between age and cancer incidence, emphasizing the need for targeted preventive measures in younger populations while ensuring robust screening and management strategies for older adults.

Based on geographic variation in incidence rates

**Figure 3:**
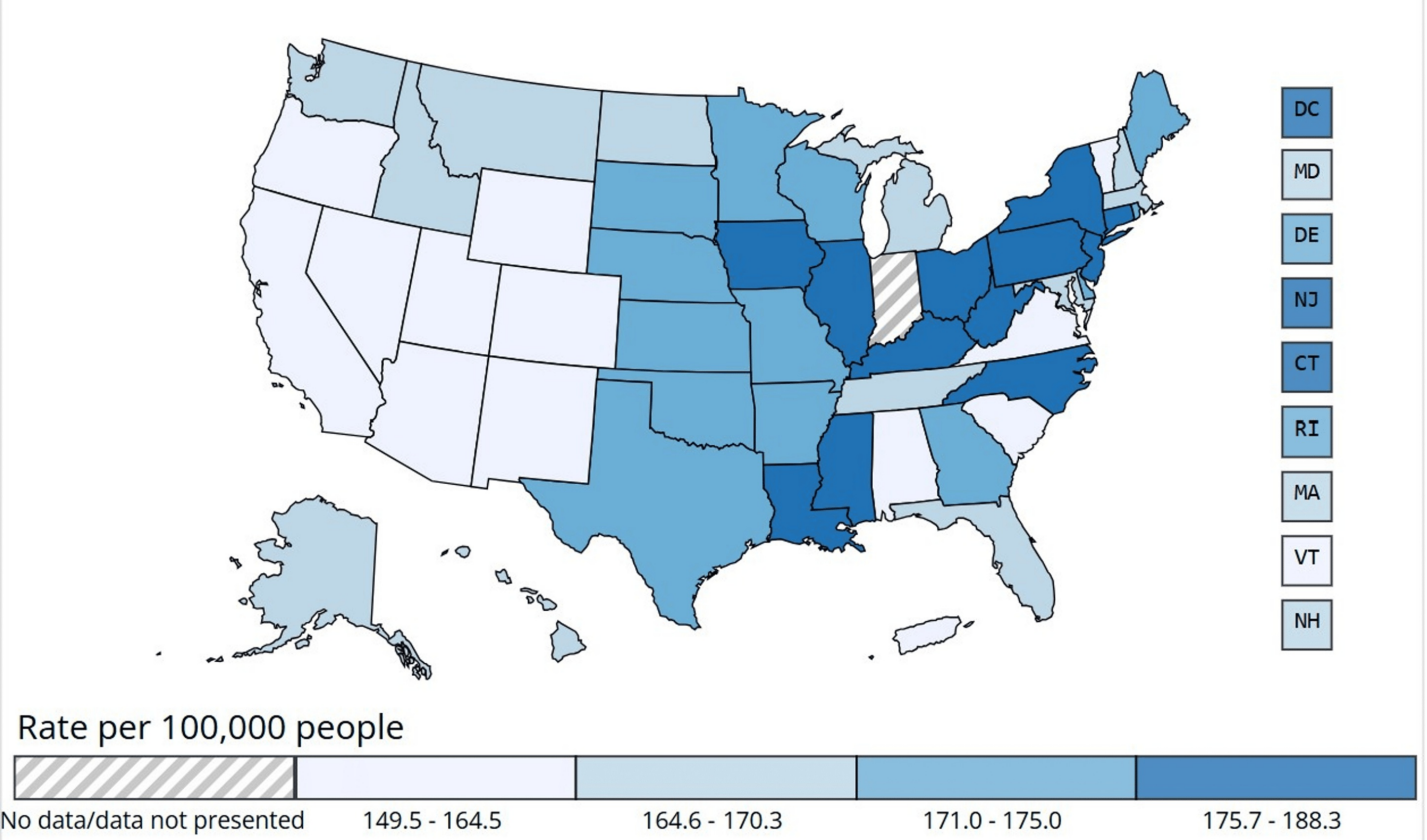
State-wise incidence rates of obesity-associated cancers in the USA Data source: Cancer data and statistics. Centers for Disease Control and Prevention (CDC) (https://www.cdc.gov/cancer/data/index.html), released in June 2024 [11].

The incidence of obesity-associated cancers varied significantly across US states from 2017 to 2021, with a national average rate of 170.0 cases per 100,000 people (95% CI: 169.8-170.2). Notable geographic disparities were observed, with some states exhibiting markedly higher incidence rates. Figure 3 illustrates the rate of new obesity-associated cancer cases per 100,000 people across different US states from 2017 to 2021, for both males and females. The data highlights geographic variations in cancer incidence, aiding in the identification of high-burden regions for targeted public health interventions. The state-wise incidence rates of obesity-associated cancers in the USA have been shown in Figure 3.

West Virginia had the highest burden, reporting an incidence rate of 188.3 per 100,000 people (95% CI: 185.8-190.8), followed by Kentucky at 186.2 (95% CI: 184.6-187.9) and Louisiana at 185.7 (95% CI: 184.0-187.3). Other states with elevated rates included Iowa (181.3, 95% CI: 179.4-183.2), Mississippi (178.7, 95% CI: 176.7-180.7), and Ohio (178.0, 95% CI: 177.0-179.0). Conversely, states with the lowest incidence rates included Nevada (149.5, 95% CI: 147.7-151.4), Arizona (153.9, 95% CI: 152.7-155.1), and Puerto Rico (154.7, 95% CI: 152.9-156.5). Other states with lower-than-average incidence included Utah (155.2, 95% CI: 153.2-157.3), Wyoming (154.7, 95% CI: 150.5-159.0), Colorado (156.7, 95% CI: 155.4-158.1), and New Mexico (158.9, 95% CI: 156.7-161.1).

A regional analysis revealed that Midwestern and Southern states exhibited the highest incidence rates, whereas the Western and Northeastern states reported lower-than-average rates. States such as California (160.0, 95% CI: 159.4-160.5), Virginia (159.9, 95% CI: 158.8-161.0), and Washington (167.6, 95% CI: 166.4-168.8) had lower rates, while Georgia (174.6, 95% CI: 173.6-175.7), Missouri (174.6, 95% CI: 173.3-176.0), and Kansas (175.0, 95% CI: 173.0-177.0) had rates exceeding the national average. These findings highlight significant geographic disparities in obesity-associated cancer incidence, emphasizing the need for targeted public health interventions in high-burden regions.

## Discussion

The findings from this study emphasize substantial disparities in obesity-associated cancer incidence across racial, age, and geographic groups. These variations reflect the complicated relationship among genetic predisposition, socioeconomic status, environmental exposures, and lifestyle behaviors in cancer risk. Addressing these disparities requires a multifaceted approach, including targeted public health interventions, equitable healthcare access, and culturally competent prevention strategies.

Our analysis revealed that Black, non-Hispanic individuals had the highest incidence of obesity-associated cancers. This aligns with previous research highlighting the disproportionate burden of obesity-related malignancies among this demographic [12]. Various factors contribute to this increased risk, including a higher prevalence of obesity, earlier onset of obesity-related complications, and barriers to accessing preventive healthcare. Additionally, structural inequities in healthcare, including delays in cancer diagnosis and treatment, exacerbate these disparities, leading to poorer outcomes in affected populations.

Similarly, American Indian and Alaska Native, non-Hispanic individuals exhibited high cancer incidence rates. This trend has been documented in prior studies. It may be influenced by a combination of factors, such as limited access to healthcare services, higher obesity prevalence, and environmental determinants of health [13]. Geographic isolation and socioeconomic hardships often restrict access to early cancer screenings and specialized oncology care, increasing the likelihood of late-stage diagnoses and poorer prognosis.

Conversely, Asian and Pacific Islander, non-Hispanic individuals demonstrated the lowest incidence of obesity-associated cancers. This is consistent with existing literature showing lower obesity prevalence in these populations [14]. Cultural dietary patterns, higher levels of physical activity, and genetic factors may contribute to their reduced cancer burden. However, despite lower overall incidence rates, disparities in cancer screening and healthcare access persist within specific Asian subpopulations, suggesting that there must be tailored public health efforts [15].

Age-specific analysis revealed a progressive increase in obesity-related cancer incidence with advancing age, peaking among individuals aged 75-79. This pattern shows that long-term obesity, problems with metabolism, and changes in cells due to aging all contribute to the development of tumors. Notably, females in this age group exhibited significantly higher incidence rates compared to males, which can largely be attributed to hormonal influences, such as increased lifetime estrogen exposure after menopause [16]. Moreover, Black, non‑Hispanic women showed particularly elevated rates, a finding that may reflect both higher obesity prevalence and disparities in healthcare access and screening uptake in this population. The high obesity prevalence among non‑Hispanic Black women reflects the combined impact of socioeconomic inequities and food deserts that limit access to healthy foods, systemic barriers to safe physical activity, and chronic psychosocial stress from racial discrimination that promotes weight gain [16]. The study on obesity-related cancers among young adults in China (2007-2021) highlights a concerning rise in incidence, demonstrating the dire need for targeted interventions. The growing cancer burden necessitates comprehensive obesity prevention strategies to mitigate future health risks [17]. Obesity increases estrogen production in adipose tissue, contributing to the development of hormone-dependent cancers such as breast and endometrial malignancies. These findings demonstrate the need for lifelong obesity prevention and early detection strategies, particularly for cancers strongly linked to hormonal imbalances [16].

Interestingly, incidence rates declined slightly among individuals aged 85 and older. This decline may be explained by survivor bias, where individuals reaching this age may have a lower cumulative cancer risk due to healthier lifestyle choices, genetic resilience, or differential mortality from competing health conditions [18]. While obesity remains a significant risk factor throughout life, these findings suggest that preventive interventions targeting younger populations may have the greatest long-term impact in reducing obesity-related cancer burden [19].

Significant geographic disparities were also observed, with states in the Midwest and South exhibiting the highest obesity-associated cancer incidence rates. States such as West Virginia, Kentucky, and Louisiana consistently reported elevated incidence, aligning with broader epidemiological trends of high obesity prevalence in these regions. Several factors contribute to these patterns, including socioeconomic disparities, lower access to healthcare services, and lifestyle behaviors such as poor dietary habits and sedentary lifestyles. The higher concentration of food deserts and limited availability of healthcare facilities in rural and economically disadvantaged areas further exacerbate these disparities [20].

In contrast, states such as Nevada and Arizona reported lower incidence rates, which may be attributed to lower obesity prevalence, greater availability of healthcare resources, and more widespread adoption of preventive health behaviors. The presence of urban centers with better healthcare infrastructure and public health initiatives focused on obesity prevention likely plays a role in mitigating cancer risk in these regions [21].

These geographic disparities highlight the critical need for region-specific public health interventions. States with high incidence rates should prioritize obesity prevention through comprehensive community-based programs that promote physical activity, healthy eating, and weight management. Expanding access to preventive healthcare services, including routine cancer screenings and obesity management programs, is essential to reducing cancer burden in high-risk populations [22].

Targeted public health interventions are crucial for addressing racial and socioeconomic disparities in obesity-associated cancer incidence. Expanding community-based initiatives that encourage healthier lifestyle behaviors can have a meaningful impact, particularly in high-burden states. Programs that focus on improving diet quality, increasing physical activity, and addressing social determinants of health can help reduce obesity prevalence and, in turn, lower cancer risk [23].

In addition to prevention, increasing access to cancer screenings, particularly for Black and American Indian populations, can aid in early detection and improve treatment outcomes. Many individuals from these communities face barriers to healthcare access, including financial constraints, lack of health insurance, and geographic obstacles. Implementing mobile screening units, expanding Medicaid coverage, and improving patient education can help bridge these gaps and promote early diagnosis [24].

Culturally tailored public health campaigns are essential for effectively addressing racial and ethnic disparities in obesity-associated cancers. Health messaging should be adapted to resonate with diverse populations, considering cultural dietary practices, traditional beliefs about health, and community-specific barriers to care. Engaging trusted community leaders and healthcare providers in outreach efforts can enhance the effectiveness of these campaigns and encourage greater participation in preventive health initiatives.

Policy-level changes also play a vital role in reducing the obesity-associated cancer burden. Addressing food deserts by improving access to affordable, nutritious foods can significantly impact obesity rates in underserved communities. Expanding healthcare access through policy reforms that reduce financial and structural barriers can ensure that individuals receive timely preventive care and cancer screenings. Additionally, implementing regulations that promote healthy environments, such as improved nutrition standards in schools and workplaces, can contribute to long-term reductions in obesity prevalence and related cancer risks [25].

The findings of this study highlight the urgency of addressing obesity-related cancer disparities through targeted interventions. Racial, age, and geographic disparities demonstrate the importance of comprehensive, evidence-based strategies that address both individual and systemic factors influencing cancer risk. By combining local programs, better access to healthcare, customized support for different cultures, and changes in policies, public health efforts can significantly help lower the rates of cancers linked to obesity and promote fair health opportunities for all groups.

Strengths and limitations

This research provides useful information about racial, age, and geographic disparities in obesity-associated cancer incidence, offering a comprehensive analysis using a large, population-based dataset. The inclusion of age-adjusted incidence rates enhances comparability across demographic groups, and the stratification by sex and geographic region strengthens the findings. Additionally, the use of a multi-year dataset (2017-2021) allows for a more robust assessment of trends, reducing the impact of short-term fluctuations. However, it is important to acknowledge certain limitations. While acknowledging inherent constraints of registry data, such as the inability to capture individual-level factors (e.g., lifestyle, socioeconomic status, genetic predisposition) and potential variation in screening practices across states and subpopulations, the critical disparities identified here warrant focused public health interventions. Future studies should build on these results by incorporating longitudinal follow-up and individual-level risk factor data to enhance understanding and optimize targeted prevention strategies.

## Conclusions

This study highlights significant racial, age, and geographic disparities in the incidence of obesity-associated cancers, emphasizing the need for targeted public health interventions. Black, non-Hispanic and American Indian/Alaska Native populations exhibited the highest incidence rates, while Asian and Pacific Islander groups had the lowest. Age-wise, cancer incidence increased with advancing age, peaking in individuals aged 75-79 years. Geographically, Midwestern and Southern states reported higher incidence rates compared to Western and Northeastern states. These findings highlight the urgent need for targeted public health interventions, including culturally tailored prevention programs, enhanced cancer screening, and policy-driven strategies to mitigate obesity and its associated cancer risks. Addressing these disparities requires a multifaceted approach that considers the unique risk factors of each population. Future research should focus on incorporating individual-level risk factors and longitudinal data to better understand the complex interactions between obesity and cancer risk, thereby refining intervention strategies and promoting equitable healthcare access.
